# Supplementary material for: Monoallelic expression and epigenetic inheritance sustained by a Trypanosoma brucei variant surface glycoprotein exclusion complex
Source: Nat Commun. 2019 Jul 9;10:3023. doi: 10.1038/s41467-019-10823-8 (PMC6617441; doi:10.1038/s41467-019-10823-8)
Supplement: Supplementary file 1 — Supplementary Information [file 41467_2019_10823_MOESM1_ESM.pdf]

Supplementary **Figures** for**:**

Monoalleic expression and epigenetic inheritance sustained by a *Trypanosoma brucei* variant surface glycoprotein exclusion complex

Joana Faria, Lucy Glover, Sebastian Hutchinson, Cordula Boehm, Mark C. Field and David Horn

This PDF file includes:

Supplementary Figures 1-8

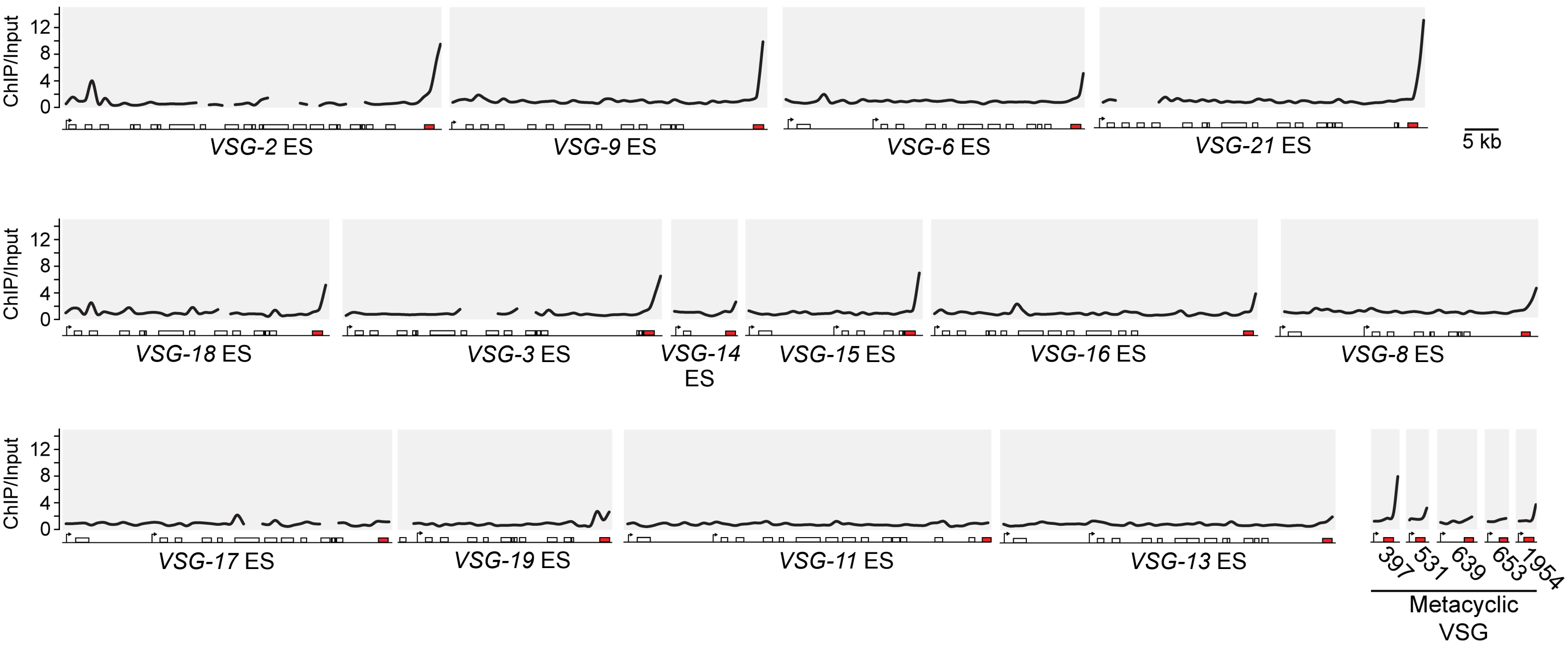

Supplementary Fig. 1. VEX1^{myc} ChIP-seq. Enrichment traces over the active and all silent *VSG*-ESs (bloodstream and metacyclic expression sites) with 1 kbp non-overlapping bins. Pol-I promoters, arrows; *ESAGs*, open boxes; *VSGs*, red boxes.

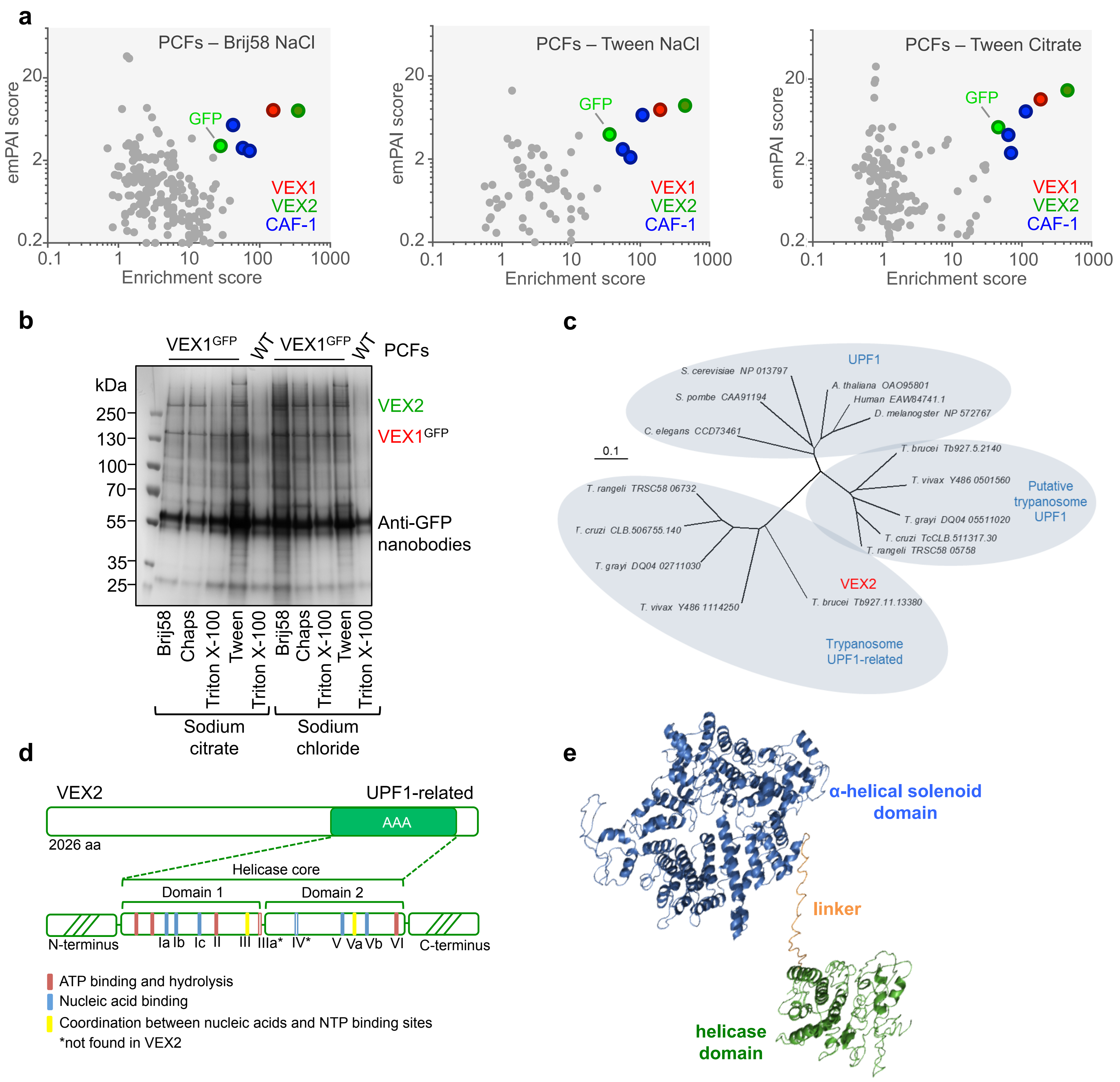

Supplementary Fig. 2. VEX1GFP affinity purification identifies VEX2, which is UPF1-related. a VEX1GFP immunoprecipitation and proteomics. GFP-tag dependent enrichment from procyclic-form *T. brucei* using various buffers. emPAI, exponentially modified protein abundance index. **b** Immunoprecipitates in a silver-stained gel. VEX1GFP, VEX2 and anti-GFP nanobodies are highlighted. All buffers contain 20 mM HEPES (pH7.4), 1 mM $MgCl_2$, 10 µM $CaCl_2$ and sodium citrate 250 mM or sodium chloride NaCl 100 mM. All detergents at 0.1%. **c** Phylogenetic analysis of the UPF1 helicases and related proteins in trypanosomatids. The unrooted neighbour-joining tree was generated using Clustal 1.8X and TreeView. **d** Conservation of the UPF1-helicase related motifs within VEX2. **e** VEX2 3D structural prediction generated using the I-Tasser platform.

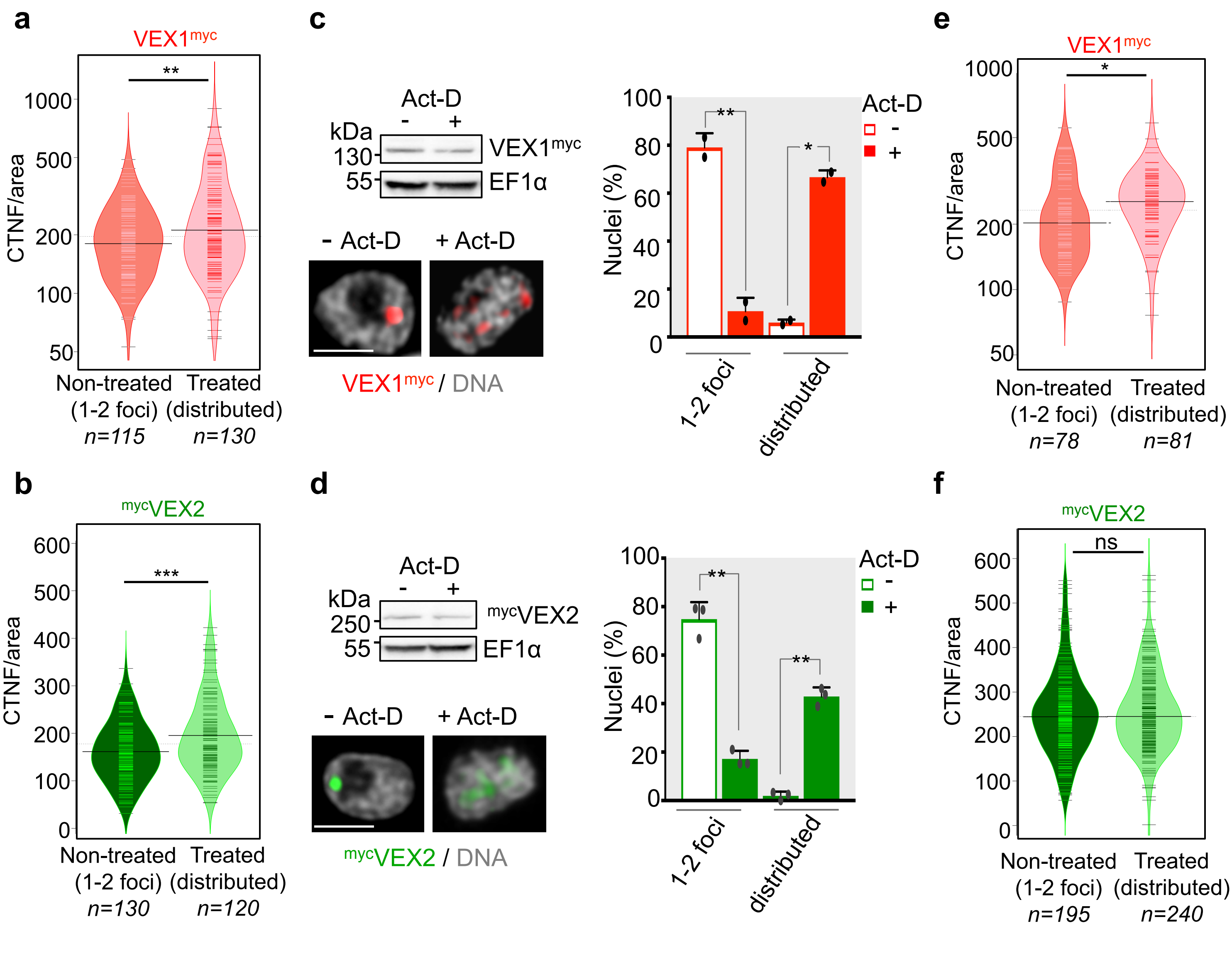

Supplementary Fig. 3. VEX1 and VEX2 localisation and signal intensity following perturbation of transcription. a-b The bean-plots indicate signal intensity in nuclei with 1-2 major VEX1^{myc} or ^{myc}VEX2 foci (non-treated) or nuclei with a distributed signal (treated with BMH-21 at 1 μM for 30 min). CTNF, corrected total nuclear fluorescence, normalized to the nuclear area. Black lines show the medians; pink/red or dark/light green lines represent individual data points; polygons represent the estimated density of the data. **c-d** Protein-blot and immunofluorescence analysis of VEX1^{myc} (c) and ^{myc}VEX2 (d) before and after actinomycin D treatment (30 min at 10 μg ml^{-1}). Proportions of nuclei displaying 1-2 major foci or distributed signals are indicated; the remaining cells displayed no detectable signal. Values are averages of two (c) or three (d) independent experiments (≥100 nuclei/each); error bars, SD. DNA was counter-stained with DAPI; the images correspond to maximal 3D projections of 30 stacks of 0.1 μm; scale bars 2 μm. **e-f** The bean-plots indicate signal intensity in nuclei with 1-2 major VEX1^{myc}/^{myc}VEX2 foci (non-treated) or nuclei with a distributed signal (treated with actinomycin D for 30 min at 10 μg ml^{-1}). Other details as in a-b. a-f are representative of independent biological replicates and independent experiments; ns, not significant; *, $p < 0.05$; **, $p < 0.01$; ***, $p < 0.001$ (two-tailed paired and unpaired Student's t-test was applied to bar graphs and bean plots, respectively). Source data are provided as a Source Data file.

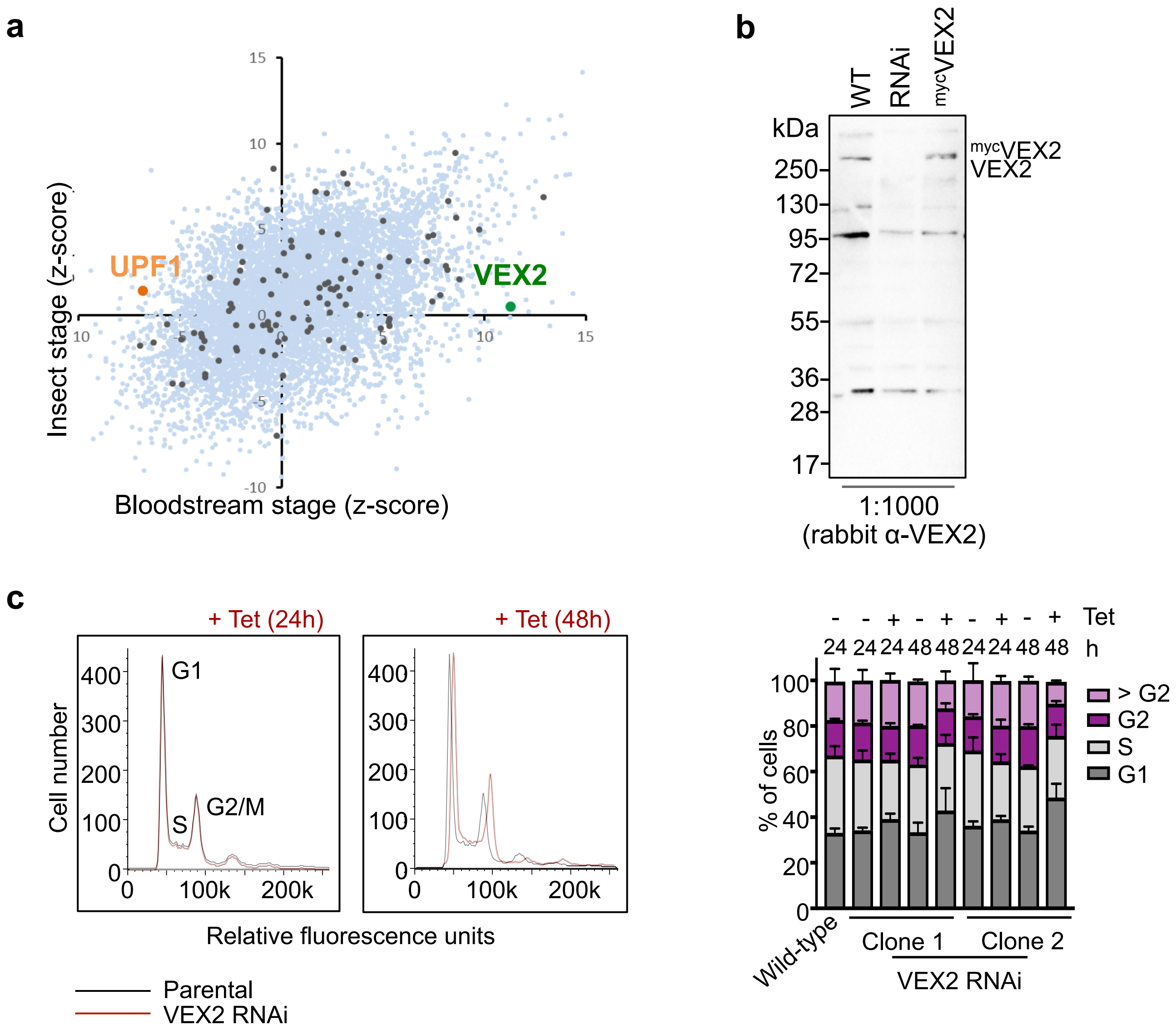

Supplementary Fig. 4. VEX2 RNAi and cell cycle analysis. a RIT-seq data analysis from genome-wide RNAi screening in both bloodstream and insect-stages (Alsford *et al*, 2011). Z-score corresponds to loss of fitness following knockdown. Thus, VEX2 registers a bloodstream-stage specific defect. Genome, grey; all DNA/RNA helicases, black; VEX2, green; canonical UPF1, orange. **b** Protein-blot analysis of wild-type, VEX2 RNAi (+Tet, 24 h) and ^{myc}VEX2 BSF cell extracts, probed with an affinity purified rabbit polyclonal α-VEX2 antibody (soluble peptide-based, custom made by Thermo Scientific). **c** Cell cycle analysis following VEX2 RNAi using propidium iodide incorporation and FACS. Representative histograms are shown on the left while the bar graph on the right indicates cells in each cell cycle stage; values are averages of two independent experiments with two technical replicates. Error bars, SD. Source data are provided as a Source Data file.

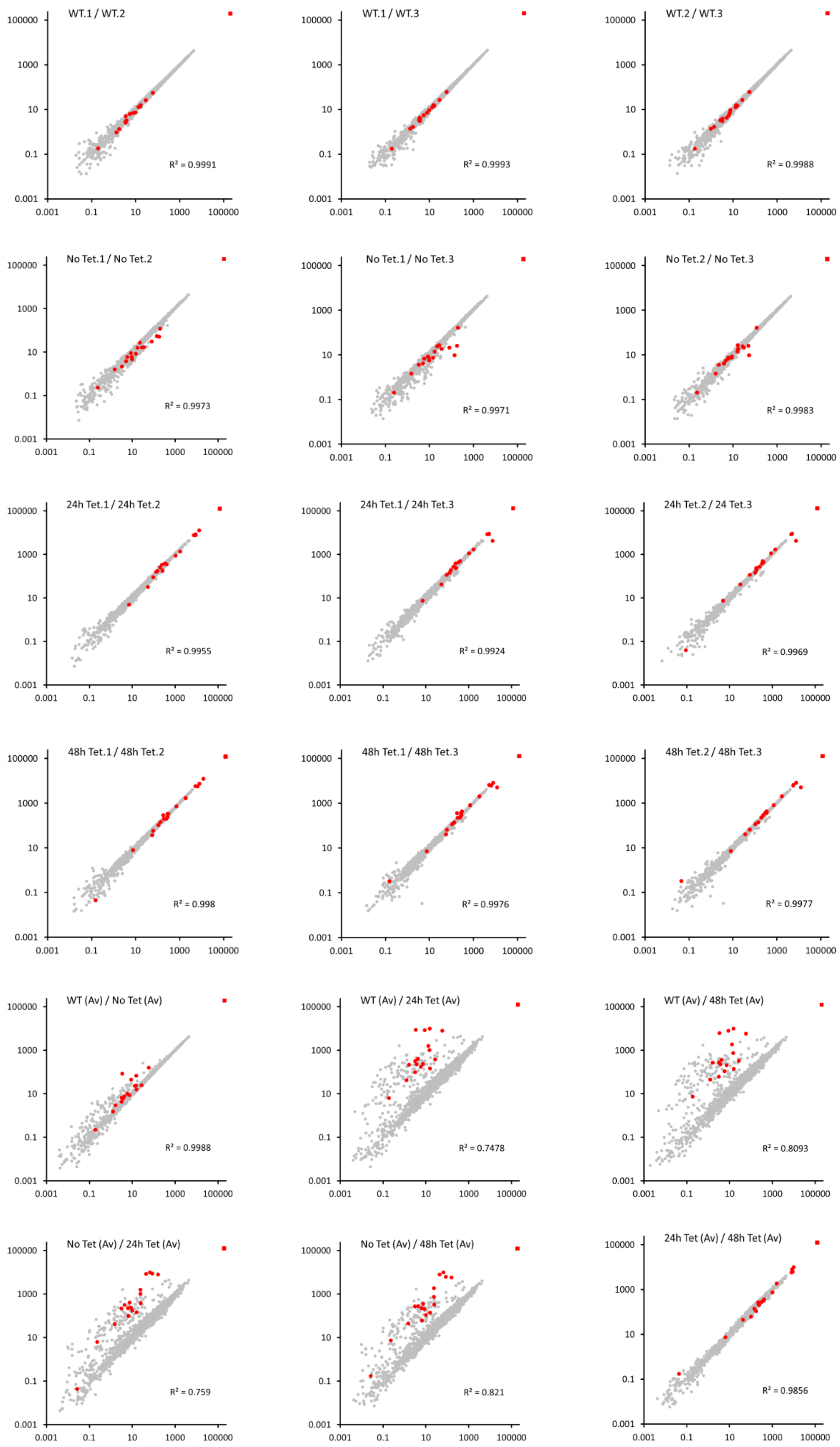

Supplementary Fig. 5. RNA-seq analysis following VEX2 knockdown. The scatter plots depict pair-wise comparisons between three biological replicates of wild-type cells and VEX2 RNAi (–Tet, +Tet 24 h, +Tet 48 h). Whole transcriptome, grey; *VSGs* from silent expression-sites, red circles; active *VSG*, red square.

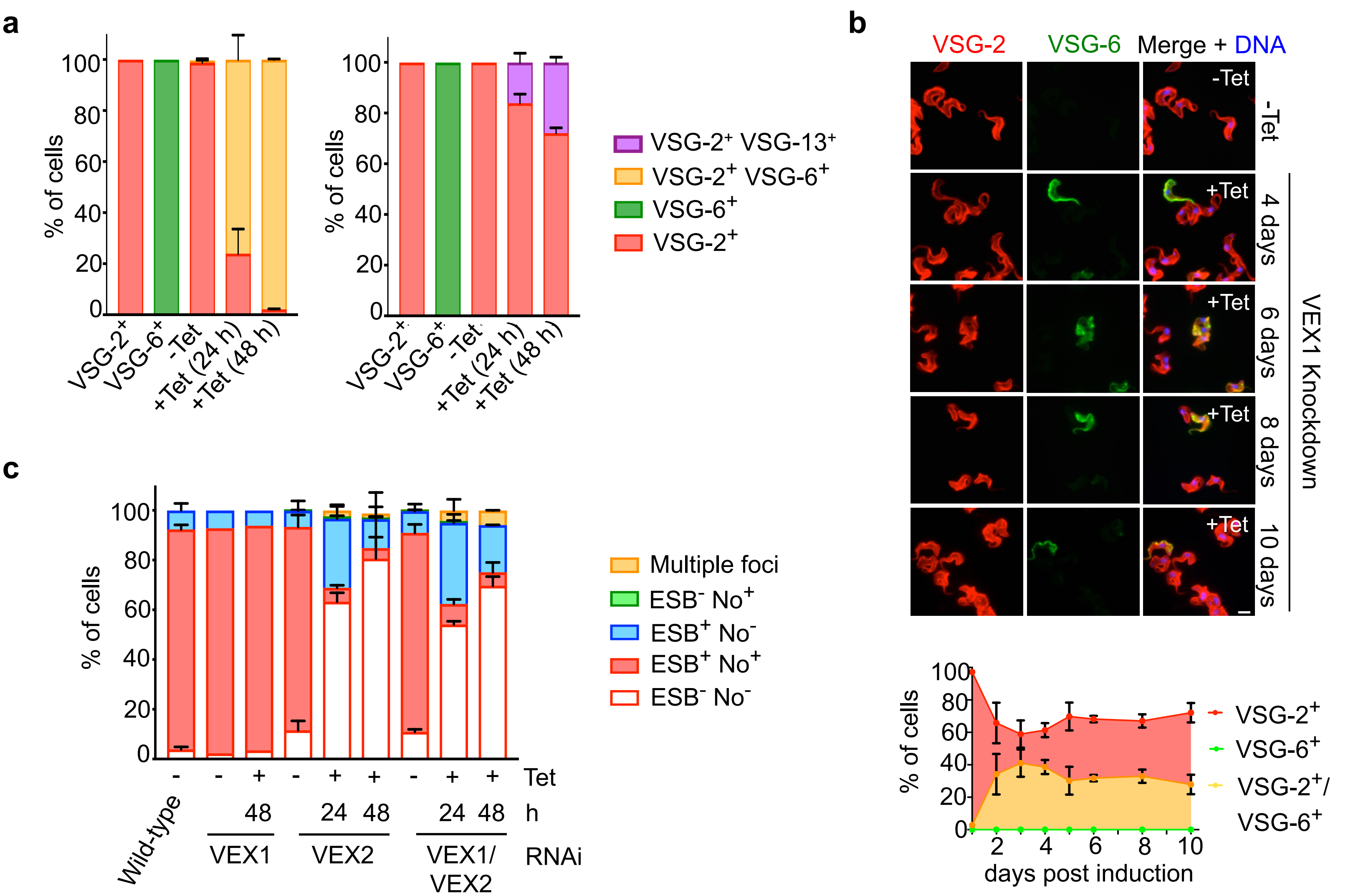

Supplementary Fig. 6. VEX1/VEX2 RNAi followed by *VSG* and pol-I analysis. a-b Quantification of dual-VSG expressers (≥100 cells) by immunofluorescence following VEX1/VEX2 (a) or VEX1 (b) RNAi. In a, cells were stained with rat anti-VSG-2 and rabbit anti-VSG-6 or rabbit anti-VSG-13. The data are averages of multiple biological replicates (a) or independent experiments (b). **c** Quantification of pol-I localization by immunofluorescence following VEX1, VEX2 or VEX1/VEX2 RNAi (≥200 nuclei). ESB, expression-site body; No, nucleolus. ESB$^-$ No$^-$ cells display non-detectable signal. Values are averages of two or three independent biological replicates. Error bars, SD. Source data are provided as a Source Data file.

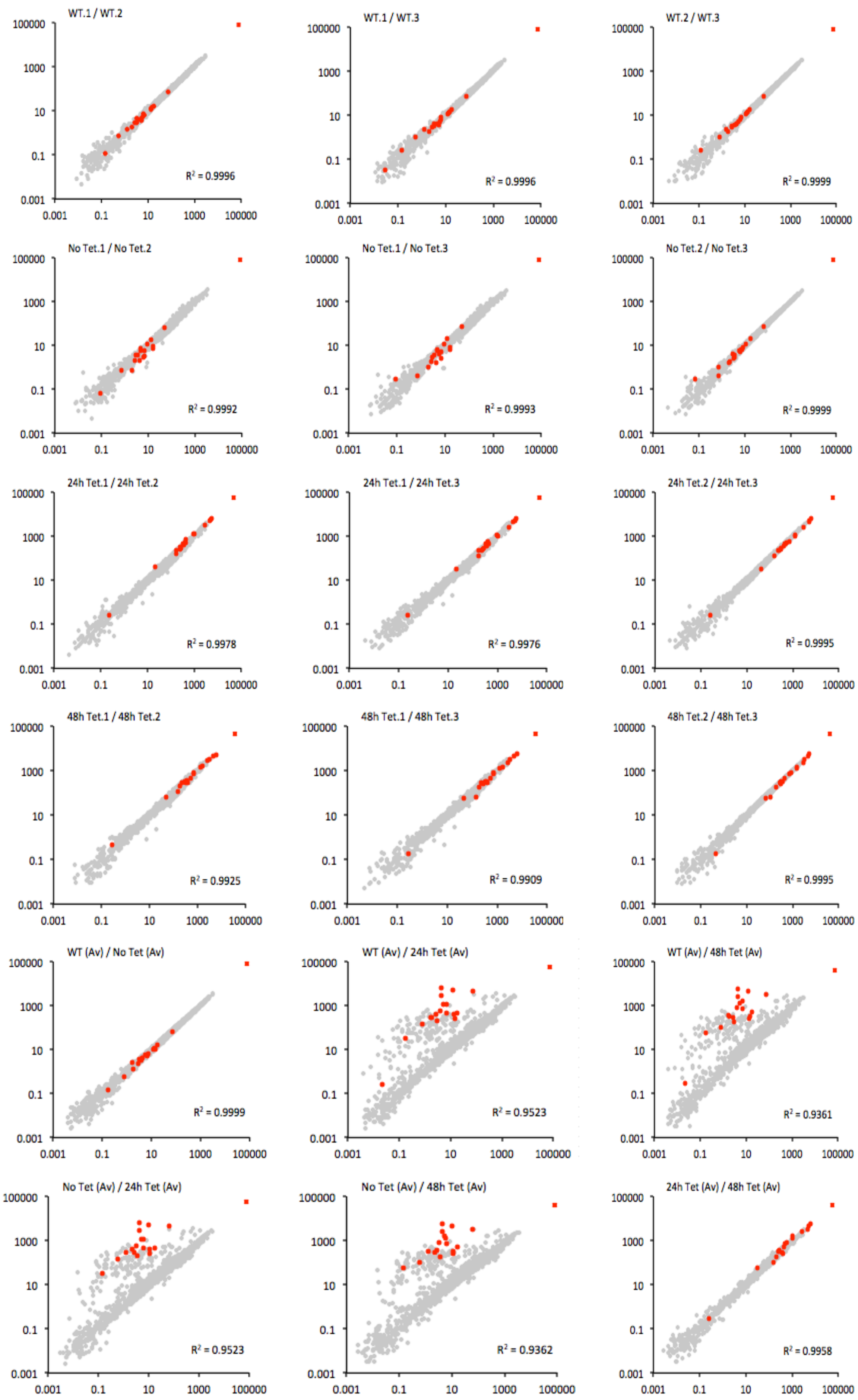

Supplementary Fig. 7. RNA-seq analysis following VEX1 and VEX2 knockdown. The scatter plots depict pair-wise comparisons between three biological replicates of wild-type cells and VEX1-VEX2 double RNAi (–Tet, +Tet 24 h, +Tet 48 h). Whole transcriptome, grey; *VSGs* from silent expression-sites, red circles; active *VSG*, red square.

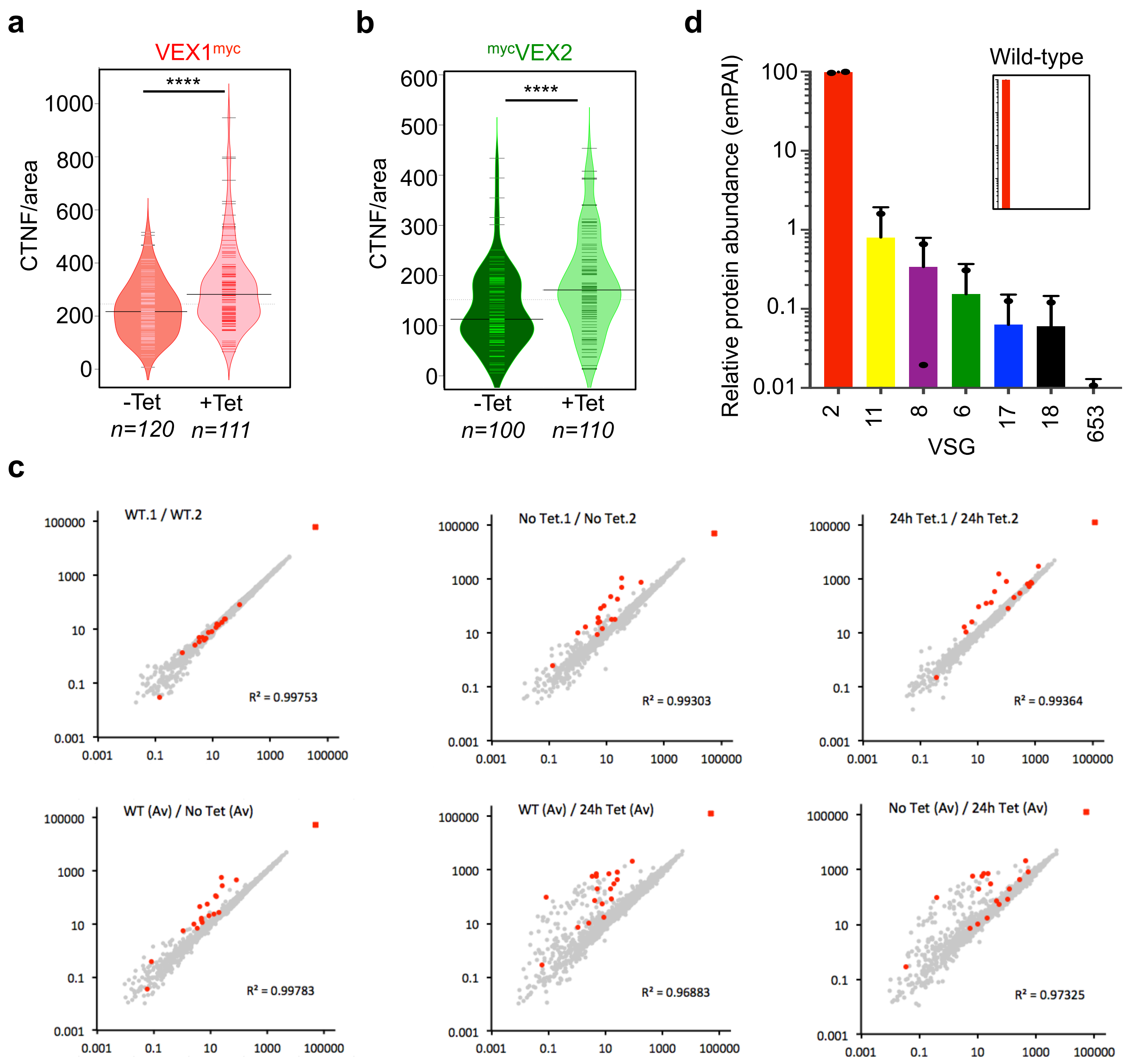

Supplementary Fig. 8. CAF-1 knockdown impacts VEX1 localisation and VSG expression. a-b The bean-plots indicate signal intensity in ≥100 nuclei with 1-2 major VEX1^{myc} (-Tet) or nuclei with a distributed signal (+Tet 12h) in a or 1-2 major ^{myc}VEX2 foci (-Tet or +Tet 12h) in b, following CAF-1b knockdown. Black lines show the medians; pink/red or dark/light green lines represent individual data points; polygons represent the estimated density of the data. CTNF, corrected total nuclear fluorescence, normalized to the nuclear area. a-b are representative of independent biological replicates and independent experiments. Error bars, SD; ****, p < 0.0001 (two-tailed unpaired Student's t-test). **c** RNA-seq following CAF-1b knockdown (24 h). The scatter plots depict pair-wise comparisons between two biological replicates of wild-type cells and CAF-1b RNAi (–Tet, +Tet 24 h). Whole transcriptome, grey; *VSGs* from silent expression-sites, red circles; active *VSG*, red square. **d** Quantitative mass spectrometry analysis of surface-VSGs following CAF-1b-knockdown (24 h). The inset shows wild-type for comparison. emPAI, exponentially modified Protein Abundance Index. The values are averages from two independent biological replicates. Source data are provided as a Source Data file.
