# Supplementary material for: Monoallelic expression and epigenetic inheritance sustained by a Trypanosoma brucei variant surface glycoprotein exclusion complex
Source: Nat Commun. 2019 Jul 9;10:3023. doi: 10.1038/s41467-019-10823-8 (PMC6617441; doi:10.1038/s41467-019-10823-8)
Supplement: Supplementary file 3 — Reporting Summary [file 41467_2019_10823_MOESM3_ESM.pdf]

nature research

Corresponding author(s): David Horn

Reporting Summary

Nature Research wishes to improve the reproducibility of the work that we publish. This form provides structure for consistency and transparency in reporting. For further information on Nature Research policies, see Authors & Referees and the Editorial Policy Checklist.

Statistical parameters

When statistical analyses are reported, confirm that the following items are present in the relevant location (e.g. figure legend, table legend, main text, or Methods section).

n/a	Confirmed	
☐	☒	The exact sample size (*n*) for each experimental group/condition, given as a discrete number and unit of measurement
☐	☒	An indication of whether measurements were taken from distinct samples or whether the same sample was measured repeatedly
☐	☒	The statistical test(s) used AND whether they are one- or two-sided<br>*Only common tests should be described solely by name; describe more complex techniques in the Methods section.*
☒	☐	A description of all covariates tested
☒	☐	A description of any assumptions or corrections, such as tests of normality and adjustment for multiple comparisons
☐	☒	A full description of the statistics including central tendency (e.g. means) or other basic estimates (e.g. regression coefficient) AND variation (e.g. standard deviation) or associated estimates of uncertainty (e.g. confidence intervals)
☐	☒	For null hypothesis testing, the test statistic (e.g. *F*, *t*, *r*) with confidence intervals, effect sizes, degrees of freedom and *P* value noted<br>*Give P values as exact values whenever suitable.*
☒	☐	For Bayesian analysis, information on the choice of priors and Markov chain Monte Carlo settings
☒	☐	For hierarchical and complex designs, identification of the appropriate level for tests and full reporting of outcomes
☒	☐	Estimates of effect sizes (e.g. Cohen's *d*, Pearson's *r*), indicating how they were calculated
☐	☒	Clearly defined error bars<br>*State explicitly what error bars represent (e.g. SD, SE, CI)*

Our web collection on statistics for biologists may be useful.

Software and code

Policy information about availability of computer code

Data collection	No previously unreported custom computer code or algorithm was central to data collection reported in the paper.
Data analysis	See 'ChIP-seq' section below. No other unreported custom computer code or algorithm used.

For manuscripts utilizing custom algorithms or software that are central to the research but not yet described in published literature, software must be made available to editors/reviewers upon request. We strongly encourage code deposition in a community repository (e.g. GitHub). See the Nature Research guidelines for submitting code & software for further information.

Data

Policy information about availability of data

All manuscripts must include a data availability statement. This statement should provide the following information, where applicable:

- Accession codes, unique identifiers, or web links for publicly available datasets
- A list of figures that have associated raw data
- A description of any restrictions on data availability

RNA-seq and ChIP-seq data have been deposited in the European Nucleotide Archive www.ebi.ac.uk/ena (accession no. PRJEB21615 and PRJEB25352, respectively). The source data underlying Figs 1d, 2a-b, 3a-e, 4f, 5a-e, 6a-c, 7a, 8a-b, 9a-c, 10a-b, Supplementary Figs 3a-f, 4c, 6a-c, 8a-b and d are provided as a Source Data file.

Field-specific reporting

Please select the best fit for your research. If you are not sure, read the appropriate sections before making your selection.

☒ Life sciences ☐ Behavioural & social sciences ☐ Ecological, evolutionary & environmental sciences

For a reference copy of the document with all sections, see nature.com/authors/policies/ReportingSummary-flat.pdf

Life sciences study design

All studies must disclose on these points even when the disclosure is negative.

Sample size	We have typically used 2 or more biological replicates, often also with technical replicates.
Data exclusions	No data were excluded from the analyses.
Replication	We typically performed multiple independent experiments.
Randomization	No randomization applied.
Blinding	No blinding applied.

Reporting for specific materials, systems and methods

Materials & experimental systems

n/a	Involved in the study
☐	☒ Unique biological materials
☐	☒ Antibodies
☐	☒ Eukaryotic cell lines
☒	☐ Palaeontology
☒	☐ Animals and other organisms
☒	☐ Human research participants

Methods

n/a	Involved in the study
☐	☒ ChIP-seq
☐	☒ Flow cytometry
☒	☐ MRI-based neuroimaging

Unique biological materials

Policy information about availability of materials

Obtaining unique materials	All the unique materials used in this study were generated by the authors and will be made available to the scientific community.

Antibodies

Antibodies used

ANTIBODY / SOURCE / IDENTIFIER
Mouse anti-Myc 9E10 / Source Bioscience / Cat# ABX298
Mouse anti-Myc 9B11 / New England Biolabs / Cat# 2276S
Mouse anti-Myc 4A6 / Merck-Millipore / Cat# 05-724 RRID:AB_568800
Mouse anti-Myc 9E10 / Abcam / Cat# Ab32
Rabbit anti-GFP / ThermoFisher Scientific / Cat# A-6455 RRID:AB_221570
Rabbit anti-GFP / Abcam / Cat# Ab290
Dimeric anti-GFP nanobodies / Inhouse production. Fridy et al, 2014
Mouse anti-EF1α CBP-KK1 / Merck-Millipore / Cat# 05-235 RRID:AB_309663
Rat anti-VSG-2 / Prof. George Cross, Rockefeller University. Hoek and Cross, 1999
Rabbit anti-VSG-2 / Prof. George Cross, Rockefeller University. Hoek and Cross, 1999
Rabbit anti-VSG-6 / Prof. George Cross, Rockefeller University. Hoek and Cross, 1999
Rabbit anti-VSG-13 / Figueiredo et al, 2008
Mouse anti-VSG-3 coupled with Alex 488 / Nicolai Siegel, LMU, Munich. Pinger et al, 2017
Rabbit anti-Histone H3 / Abcam / Cat# Ab1791 RRID:AB_302613
Rabbit anti-NOG1 / Park et al, 2001
Mouse anti-EP procyclin / VWBio-Cedarlene / Cat# CLP001AP RRID:AB_10060662
Rabbit anti-Pol-I / Glover et al, 2016.

Mouse anti-EP procyclin / VWBio/Cedarlene / Cat# CLP001AP RRID:AB_10060662
Rabbit anti-VEX2 / This paper, custom made by Thermo Scientific.
Goat anti-mouse Alexa 488 / ThermoFisher Scientific / Cat# A-11001 RRID:AB_2534069
Goat anti-rabbit Alexa 488 / ThermoFisher Scientific / Cat# A-11034 RRID:AB_2576217
Goat anti-mouse Alexa 568 / ThermoFisher Scientific / Cat# A-11004 RRID:AB_2534072
Goat anti-rabbit Alexa 568 / ThermoFisher Scientific Cat# A-11011 RRID:AB_143157
Goat anti-rat Alexa 647 / ThermoFisher Scientific / Cat# A-21247 RRID:AB_141778
Chicken anti-rat Alexa 488 / ThermoFisher Scientific / Cat# A-21470 RRID:AB_2535873
Goat anti-mouse HRP / Biorad / Cat# 1721011 RRID:AB_11125936
Goat anti-rabbit HRP / Biorad / Cat# 1706515 RRID:AB_11125142

Validation

There was one antibody specifically produced for this study (rabbit polyclonal anti-VEX2), it was used for protein-blot analysis only, and not for IFA, due to the lack of specificity. It was validated for protein-blot by using a cell line where the protein was specifically depleted.

Eukaryotic cell lines

Policy information about cell lines

Cell line source(s)

Trypanosoma brucei brucei Lister 427 was originally obtained from Prof. George Cross (Rockefeller University, NYC, USA). Subsequent genetic modifications were performed by the authors.

Authentication

RNA-seq provided authentication.

Mycoplasma contamination

Mycoplasma contamination check carried our approx. every 3 years - no positive results from those tests to date.

Commonly misidentified lines
(See ICLAC register)

T. b. brucei L427 is not a commonly misidentified line.

ChIP-seq

Data deposition

☒ Confirm that both raw and final processed data have been deposited in a public database such as GEO.

☒ Confirm that you have deposited or provided access to graph files (e.g. BED files) for the called peaks.

Data access links
May remain private before publication.

www.ebi.ac.uk/ena (accession no. PRJEB25352).

Files in database submission

Fastq and BAM files

Genome browser session
(e.g. UCSC)

No longer applicable

Methodology

Replicates

One experiment: ChIP v input. All known telomeric VSGs (n=19) and ESAGs (n=129) were scored. The active VSG was the most enriched gene among this full set (n=148).

Sequencing depth

54.0 and 49.9 million read pairs were aligned.

Antibodies

Mouse anti-Myc 9E10 / Source Bioscience / Cat# ABX298

Peak calling parameters

Heat maps were generated using deeptools2 and resulting vector graphics files were then assembled into figures using Adobe Illustrator. Telomeric VSG coding sequences with a mapping quality filtering of >=2 were aligned using deeptools computeMatrix scale-regions using the following commands:

```
$ bamCompare -b1 BSF-VEX1-12M-CHIP_mkdup.bam \
-b2 BSF-VEX1-12M-INPUT_mkdup.bam \
-o BSF-VEX1-12M-ChIP_InputSpf1_CellI_mkdup_bs10b_smooth_len30_mapQ2.bw \
-bs 10 -p 4 --smoothLength 30 -of bigwig --minMappingQuality 2

$ computeMatrix scale-regions -S BSF-VEX1-12M-ChIP_InputSpf1_CellI_mkdup_bs10b_smooth_len30_mapQ2.bw \
-R Tbrucei927_427telomeres_mVSG_spf1__Cellii_TelomericVSG_BED.bed \
-b 1000 -a 1000 -bs 10 -p 4 -out ALL_ChIP_Log2_ab1000_bs10-sl30_VSGs_mapQ2 --sortRegions keep

$ plotHeatmap -m ALL_ChIP_Log2_ab1000_bs10-sl30_VSGs_mapQ2 \
—outFileNameMatrix ALL_ChIP_Log2_ab1000_bs10-sl30_VSGs.txt \
-o ALL_ChIP_Log2_ab1000_bs10-sl30_teloVSGs.svg \
—yMin -1 --yMax 6 --heatmapHeight 5 --zMin -5 --zMax 5 --dpi 300 --sortRegions no\
—outFileSortedRegions ALL_ChIP_Log2_ab1000_bs10-sl30_VSGs_sortedregions.txt
```

For ESs, reads were counted using bedtools, counting reads in non-overlapping 1 kb bins. All bedgraph files were imported

	into Microsoft Excel and plotted. Files were normalised by counting total read counts per library prior to fold change calculation for each bin. These were then plotted and assembled in Illustrator. Locus maps were generated by exporting vector graphics views or regions of interest from Artemis genome browser and manipulated in Adobe illustrator.
Data quality	Reads were aligned using bowtie2 in very-sensitive alignment mode and alignments compressed and sorted using samtools. PCR duplicate reads were removed using Picard MarkDuplicates.
Software	Bowtie2<br>Samtools<br>deeptools2<br>Picard tools<br>BEDtools<br>Artemis<br>RStudio<br>EdgeR

Flow Cytometry

Plots

Confirm that:

☒ The axis labels state the marker and fluorochrome used (e.g. CD4-FITC).

☒ The axis scales are clearly visible. Include numbers along axes only for bottom left plot of group (a 'group' is an analysis of identical markers).

☒ All plots are contour plots with outliers or pseudocolor plots.

☒ A numerical value for number of cells or percentage (with statistics) is provided.

Methodology

Sample preparation	Cells were fixed and stained for VSGs and PI.
Instrument	BD LSRFortessa (BD Biosciences)
Software	FlowJo software
Cell population abundance	> 40,000 events were analysed to determine the percentage of cells in each quadrant.
Gating strategy	The cells were first gated using FSC and SSC, then gated using the PI staining, and only then Alexa488 versus Alexa647.

☒ Tick this box to confirm that a figure exemplifying the gating strategy is provided in the Supplementary Information.
