# Supplementary material for: Monoallelic expression and epigenetic inheritance sustained by a Trypanosoma brucei variant surface glycoprotein exclusion complex
Source: Nat Commun. 2019 Jul 9;10:3023. doi: 10.1038/s41467-019-10823-8 (PMC6617441; doi:10.1038/s41467-019-10823-8)
Supplement: Supplementary file 4 — Description of Additional Supplementary Files [file 41467_2019_10823_MOESM4_ESM.pdf]

**Description of Additional Supplementary Files**

File Name: Supplementary Data 1
Description:
Sheet 1. ChIP-seq data for VEX1$^{myc}$ expressing bloodstream form T. brucei.
Sheet 2. Proteomic data for GFP-tag associated enrichment from VEX1$^{GFP}$ expressing insect and bloodstream form T. brucei.
Sheet 3. Proteomic analysis of purified sVSG fraction following VEX2, VEX1/VEX2 or CAF-1b knockdown.
Sheet 4. RNA-seq data following VEX2, VEX1/VEX2 or CAF-1b knockdown. Specifically showing VSG derepression (RPKM averages).
Sheet 5. RNA-seq data following VEX2, VEX1/VEX2 or CAF-1b knockdown (RPKM values).
Sheet 6. Summary of all statistical analyses.
Sheet 7. Reagents and resources.

File Name: Supplementary Movie 1
Description: 3D projection of T. brucei cell showing VEX1$^{Myc}$ (red) and VEX2$^{GFP}$ (green). Brighter grey/white region is the mitochondrial genome

File Name: Supplementary Movie 2
Description: 3D projection of late S-phase T. brucei cell showing CAF-1b$^{GFP}$ (green) colocalisation with VEX1$^{Myc}$ (red). Brighter grey/white region is the mitochondrial genome.
